# Reliability of the Luganda version of the Child Behaviour Checklist in measuring behavioural problems after cerebral malaria

**DOI:** 10.1186/1753-2000-3-38

**Published:** 2009-12-08

**Authors:** Paul Bangirana, Noeline Nakasujja, Bruno Giordani, Robert O Opoka, Chandy C John, Michael J Boivin

**Affiliations:** 1 Department of Psychiatry, Makerere University School of Medicine, Kampala, Uganda; 2 Department of Public Health Sciences, Karolinska Institutet, Stockholm, Sweden; 3 Neuropsychology Section, Department of Psychiatry, University of Michigan, Ann Arbor, Michigan, USA; 4 Department of Pediatrics and Child Health, Makerere University School of Medicine, Kampala, Uganda; 5 Department of Pediatrics, University of Minnesota, Minneapolis, Minnesota, USA; 6 International Neurologic and Psychiatric Epidemiology Program, Michigan State University, East Lansing, Michigan, USA

## Abstract

**Background:**

No measure of childhood behaviour has been validated in Uganda despite the documented risks to behaviour. Cerebral malaria in children poses a great risk to their behaviour, however behavioural outcomes after cerebral malaria have not been described in children. This study examined the reliability of the Luganda version of the Child Behaviour Checklist (CBCL) and described the behavioural outcomes of cerebral malaria in Ugandan children.

**Methods:**

The CBCL was administered to parents of 64 children aged 7 to 16 years participating in a trial to improve cognitive functioning after cerebral malaria. These children were assigned to the treatment or control group. The CBCL parent ratings were completed for the children at baseline and nine weeks later. The CBCL was translated into Luganda, a local language, prior to its use. Baseline scores were used to calculate internal consistency using Cronbach Alpha. Correlations between the first and second scores of the control group were used to determine test-retest reliability. Multicultural norms for the CBCL were used to identify children with behavioural problems of clinical significance.

**Results:**

The test-retest reliability and internal consistency of the Internalising scales were 0.64 and 0.66 respectively; 0.74 and 0.78 for the Externalising scale and 0.67 and 0.83 for Total Problems. Withdrawn/Depressed (15.6%), Thought Problems (12.5%), Aggressive Behaviour (9.4%) and Oppositional Defiant Behaviour (9.4%) were the commonly reported problems.

**Conclusion:**

The Luganda version of the CBCL is a fairly reliable measure of behavioural problems in Ugandan children. Depressive and thought problems are likely behavioural outcomes of cerebral malaria in children. Further work in children with psychiatric diagnoses is required to test its validity in a clinical setting.

## Background

Mental and behavioural problems in children and adolescents are common with 10-20% of the world's children estimated to have one or more such problems [1]. These high rates of behavioural problems in children and adolescents are attributed to several factors including poverty, armed conflict, infectious diseases like malaria and HIV/AIDS, unfavourable family environments and substance abuse [1-5]. In some cases of exposure to war trauma, the percentage of those with symptoms of clinical importance can range from 50 to 90% [2].

In Uganda, malaria accounts for 30% of paediatric admissions with approximately 6500 admissions annually at Mulago Hospital, the National Referral Hospital [6]. Cerebral malaria accounts for 8.2% of these malaria cases with a mortality of 17% [6]. Despite its low prevalence, cerebral malaria is one of the major causes of neurodevelopmental difficulties [7,8] with several studies documenting cognitive and neurologic deficits in survivors, with 14% to 26% having cognitive deficits [9-11]. There is however little evidence of behavioural problems resulting from cerebral malaria with only one study describing the behavioural problems [12]. The other studies that have assessed the behavioural outcomes of cerebral malaria are either cases studies [13,14] with limited ability to generalise, or do not describe the behavioural functions affected [15,16]. Documentation of the behavioural problems after cerebral malaria can highlight what problems to assess for in survivors and to help develop appropriate interventions for affected children. Despite the lack of documentation of behavioural problems, the burden of cerebral malaria on children's development is evident with estimates putting the number of children with cognitive and behavioural problems after cerebral malaria at over 200,000 per year [11,16].

In order to deal with the increasing burden of mental and behavioural problems, WHO has emphasised the need to manage and treat patients in primary health centres [1]. In low and middle-income countries where there is a shortage of trained mental health professionals [3,4,17], there may be difficulties in correctly assessing, diagnosing and treating mental and behavioural problems. In Uganda for example, 36% of physically ill adult patients seeking treatment at primary health care facilities have a current major depressive episode [18]. Due to the large patient load and health workers not suspecting mental illness as the underlying problem [19], patients may only be treated for the presenting problem (e.g., backaches, fevers), leaving the psychiatric problem causing these complaints untreated.

One way to overcome the difficulty in patient management is to use psychiatric rating scales that are validated in the target population and summarise the patients' complaints into a probable psychiatric diagnosis. These scales can be completed by a child's caregiver as a screening tool and can also track treatment progress. These rating scales also ensure appropriate investigations and treatment, thus saving time and limited resources in suspected psychiatric cases. In non-clinical settings, ratings scales for children have been used in assessing the functioning of immigrant children [20].

One such childhood rating scale is the Child Behaviour Checklist (CBCL), a widely used psychiatric rating scale for children and adolescents [21]. Research with the CBCL has demonstrated its sound reliability and validity for the scale in different cultures [22-24], and cross-cultural norms have been established [20]. A recent validation of the school age CBCL in 30 societies showed that its eight syndrome scales have a good fit when tested separately in these societies and that they are a reasonable tool for conceptualising children's emotional and behavioral difficulties in those societies [22].

Despite its sound reliability and validity in a number of cultures, the CBCL is yet to be validated in Uganda. We present a study carried out in Ugandan children to examine the reliability of the Luganda version of the CBCL and to document the behavioural problems after cerebral malaria as measured by the CBCL.

## Methods

### Study population and recruitment

The present study was conducted at Mulago Hospital, Kampala, Uganda from November 2007 to April 2008. Study participants were a cohort of cerebral malaria survivors earlier admitted to the hospital who participated in studies examining the cognitive and neurological outcomes of the disease with testing at 0, 3, 6, and 24 months [11,25]. The children were recruited into these earlier studies if they were admitted to Mulago Hospital and met the WHO criteria for cerebral malaria namely, coma (Blantyre Coma Scale score of ≤ 2 or Glasgow Coma Scale score of ≤ 8), *Plasmodium falciparum *on blood smears, and no other cause of coma. Of the 86 children enrolled in these earlier studies, 65 were traced after the 24 months assessments and invited to participate in a pilot clinical trial to improve cognition in children surviving cerebral malaria [26]. Only children enrolled in this clinical trial were included in the current study. Thirty two of the 65 children were assigned to sixteen 45 minute computerized cognitive rehabilitation therapy (CCRT) sessions over 8 weeks and the other 33 to the control arm. One child in the control arm died before completing post-intervention assessment. Of the 65 children included into the intervention study [26], one in the control arm was excluded because the CBCL was administered in English leaving 64 children for inclusion in this study.

Written informed consent and assent was obtained from the parents/guardians and the children. Ethical approval for this study was granted by the Institutional Review Boards for Human Studies at Makerere University School of Medicine, Michigan State University, University of Michigan and the Uganda National Council for Science and Technology.

### Assessments

This CBCL is a paper-pencil child behavioural assessment consisting of 120 items to which a parent/guardian responds. These items can be categorised into eight syndrome scales (Anxious/Depressed, Withdrawn/Depressed, Somatic Complaints, Social Problems, Thought Problems, Attention Problems, Rule-Breaking Behaviour and Aggressive Behaviour) or six Diagnostic and Statistical Manual (DSM) oriented scales (Affective Problems, Anxiety Problems, Somatic Problems, Attention Deficit/Hyperactivity, Oppositional Defiant Problems and Conduct Problems). The items can further be summarised into Internalising Problems (summation of Anxious/Depressed, Withdrawn/Depressed and Somatic Complaints scales), Externalising Problems (summation of Rule-Breaking Behaviour and Aggressive Behaviour scales) or into one summary score; Total Problems (summation of all items). The eight Syndrome scales have been validated in 30 societies and have proven useful in multicultural assessment of children [22].

From the 30 societies above, three groups of CBCL scores have been developed corresponding to low, medium or high problems [20]. These groups each give cut-offs for boys and girls in the age ranges 6-11 and 12-18 showing which scores are normal, borderline or of clinical significance. This study utilised scores from Group 3 (comprising of CBCL scores from Algeria, Ethiopia, Portugal and Puerto Rico) which are higher than the other two groups indicating more behavioural problems. Group 3 was chosen because the countries in this group are more similar to Uganda than countries in the other groups. Scores equal or higher than the lowest score in clinical range of the Group 3 norms were categorised as being of clinical significance. The cutoffs for scores in the clinical range were at the 97% percentile [21] suggesting that 3% of the Group 3 sample had behavioural problems.

Prior to its use, the CBCL was translated into Luganda by a research assistant and then back-translated to English by another research assistant, both fluent in Luganda and English. The second author (NN), a Psychiatrist fluent in both Luganda and English compared the two English versions and resolved any discrepancies by editing the translated version to match the original English version. However, the translation was not checked nor authorized by the authors of the CBCL.

### Procedures

Children were traced from records of two studies looking at the cognitive and neurologic outcomes of cerebral malaria in which they participated [11,25] and given appointments to return to the study clinic. Consent and assent were sought from the parent/guardian and child respectively. Thirty two of the children were assigned to the control group and the other 32 to the cognitive rehabilitation intervention group by use of random numbers. While the child completed cognitive testing as part of the trial to treat cognitive deficits after cerebral malaria [26], research assistants fluent in Luganda administered the CBCL to the mother. Repeat testing for the control group with the CBCL was done nine weeks later to determine the test-retest reliability. Scores from the baseline testing were then compared to the Group 3 norms to identify scores that are of clinical significance.

Out of the 32 controls, 10 (31.3%) assessments were done with a respondent who doesn't spend much time with the child compared to 8 (25%) in the intervention group. In the test-retest analyses, these 10 CBCLs were excluded.

### Statistical analysis

Data was analysed using SPSS 16. Variables that were not normally distributed were log-transformed prior to analysis. Test-retest reliability of the CBCL's 17 scales was assessed by running Pearson's correlations between baseline and follow up scores while the internal consistency was assessed by running Cronbach's Alpha coefficient on the baseline scores. The test-retest reliability analyses were done on the control group only while the internal consistency analyses were carried out on the baseline scores of the whole sample. The intervention group was excluded from the test-retest reliability because the cognitive rehabilitation training can improve behavioural scores [26], which could in turn affect the correlation between the pre- and post-intervention ratings by parents for the intervention group. Cross tabulations were used to compare the frequency of behavioural problems of clinical significance between the sexes. The means and standard deviations for the different sexes and age groups were averaged to present the overall score of the Ugandan children in the different CBCL scales. Cohen's *d *was then calculated [27,28] to compare how much Ugandan and Group 3 children's scores differed.

## Results

### Demographic characteristics

The mean age of the study children was 9.88 years (SD = 2.47) with a preponderance for males (60.90%). The mean years spent in school was 3.86 (SD = 2.31). Table 1 presents detailed demographic characteristics of the study children.

**Table 1:** Participants' demographic characteristics

Domain	Number (%)	M (SD)
	N = 64	
Gender, male	39 (60.9)	
Age (years)		9.88 (2.47)
School grade		3.86 (2.31)
Weight (kgs)		28.71 (8.95)
Height (cm)		131.24 (15.68)
Weight for z score (WAZ)		-0.90 (0.85)
Interval between pre- and post-testing (days)		71.28 (16.14)

WAZ; Weight for age z score.

### Reliability of the CBCL

The test-retest reliability of the Internalising, Externalising and Total Problems Scales were 0.64, 0.74 and 0.67 respectively. The reliabilities for the other Syndrome and DSM Scales ranged from 0.82 (Aggressive Behaviour) to 0.19 (Thought Problems).

The internal consistency of the Internalising, Externalising and Total Problems Scales were 0.66, 0.78 and 0.83 respectively. The internal consistency of the other scales ranged from 0.70 (Aggressive Behaviour) to 0.24 (Thought Problems). See Table 2.

**Table 2:** Test-retest reliability and internal consistency of the CBCL

CBCL syndrome scales	Test-retest reliability N = 22	Internal consistency N = 64
1. Anxious/Depressed	0.35	0.53
2. Withdrawn/Depressed	0.73**	0.62
3. Somatic Complaints	0.74**	0.50
4. Social Problems	0.73**	0.41
5. Thought Problems	0.19	0.24
6. Attention Problems	0.64**	0.57
7. Rule-Breaking Behaviour	0.53**	0.51
8. Aggressive Behaviour	0.82**	0.70
9. Internalising Problems	0.64**	0.66
10. Externalising Problems	0.74**	0.78
11. Total Problems	0.67**	0.83

**CBCL DSM scales**		

1. Affective Problems	0.45*	0.44
2. Anxiety Problems	0.62**	0.33
3. Somatic Problems	0.69**	0.36
4. ADH Problems	0.65**	0.61
5. Oppositional Behaviour	0.70**	0.58
6. Conduct Problems	0.57**	0.69

*p < 0.05, **p < 0.001, CBCL: Child Behaviour Checklist, DSM: Diagnostic and Statistical Manual for Mental Disorders

### Frequency of behavioural problems of clinical significance

Table 3 presents the frequency of behavioural problems with the actual total count for observed problems given in the second column (with the percentage in parentheses) and further presented by sex and age group in the next columns. Withdrawn/Depressed problems were commonly reported by the parents/caretakers with 15.6% followed by Thought Problems at 12.5%, Aggressive Behaviour at 9.4% and Oppositional Behaviour at 9.4%. Attention Problems at 0% and Rule-Breaking Behaviour at 1.6% were the least reported. On the Total Problems score, 14.1% had a score of clinical significance while 31.3% had Internalising Problems and 23.4% with Externalising Problems. Though more females were reported with Conduct Problems than males, this difference would not be significant when corrected for the number of analyses. No other differences were observed in the frequency and severity of behavioural problems between the sexes.

**Table 3:** Frequency of behavioural problems after cerebral malaria

Domain	Total sample^1^ N = 64		Between Sexes		Between age groups	
			Male	Female	6-11	12-18
**CBCL syndrome scales**	**N**	**%**	**N**	**N**	**N**	**N**
1. Anxious/Depressed	3	4.7	2	1	2	1
2. Withdrawn/Depressed	10	15.6	8	2	7	3
3. Somatic Complaints	3	4.7	3	0	1	2
4. Social Problems	4	6.3	2	2	3	1
5. Thought Problems	8	12.5	4	4	8	0
6. Attention Problems	0	0	0	0	0	0
7. Rule-Breaking Behaviour	1	1.6	0	1	1	0
8. Aggressive Behaviour	6	9.4	2	4	5	1
9. Internalising Problems	20	31.3	14	6	15	5
10. Externalising Problems	15	23.4	6	9	13	2
11. Total Problems	9	14.1	3	6	8	1

**CBCL DSM scales**						

1. Affective Problems	5	7.8	4	1	4	1
2. Anxiety Problems	3	4.7	3	0	3	0
3. Somatic Problems	5	7.8	4	1	3	2
4. ADH Problems	3	4.7	2	1	3	0
5. Oppositional Behaviour	6	9.4	2	4	5	1
6. Conduct Problems	4	6.3	0*	4*	3	1

* < 0.05; Fisher's exact test used for all between group comparisons. ^1^Number and frequency of children with behavioural problems.

### Comparison with the Group 3 norms

Ugandan children had higher scores on behavioural problems than the Group 3 parents for all the scales (as indicted by Cohen's *d *scores greater than zero) except attention problems (Table 4). Scores for Total Problems, Internalising Problems and Aggressive Behaviour were the most deviant from the Group 3 sample.

**Table 4:** Comparison of Ugandan scores with Group 3 norms of the CBCL

CBCL syndrome scales	Uganda M (SD)	Group 3 M (SD)	Cohen's *d*
1. Anxious/Depressed	8.0 (3.2)	4.9 (3.4)	1.66
2. Withdrawn/Depressed	6.5 (3.8)	4.2 (3.0)	1.32
3. Somatic Complaints	4.3 (1.4)	2.9 (2.7)	0.89
4. Social Problems	5.9 (1.6)	3.7 (3.0)	1.32
5. Thought Problems	4.8 (1.4)	2.2 (2.5)	1.65
6. Attention Problems	4.6 (2.0)	5.8 (4.3)	-0.58
7. Rule-Breaking Behaviour	3.6 (2.3)	3.5 (3.6)	0.05
8. Aggressive Behaviour	13.2 (3.4)	6.9 (5.6)	2.65
9. Internalising Problems	18.8 (3.4)	9.5 (7.3)	3.48
10. Externalising Problems	16.7 (5.6)	10.3 (8.3)	2.22
11. Total Problems	57.6 (10.8)	38.2 (23.0)	4.07

**CBCL DSM scales**			

1. Affective Problems	6.2 (1.7)	4.1 (3.1)	1.25
2. Anxiety Problems	3.1 (0.8)	2.7 (2.0)	0.28
3. Somatic Problems	3.0 (1.1)	1.8 (1.9)	0.92
4. ADH Problems	4.6 (1.5)	4.7 (3.3)	-0.07
5. Oppositional Behaviour	4.5 (1.3)	2.8 (2.2)	1.17
6. Conduct Problems	4.8 (3.2)	3.1 (3.3)	0.95

## Discussion

This study was carried out to examine the reliability of the Luganda version of the CBCL and document behavioural problems in a sample of Ugandan children with a history of cerebral malaria. Test-retest reliabilities and internal consistencies for the three main scales (Internalising Problems, Externalising Problems and Total Problems) ranged from 0.64 to 0.83. Other studies of the CBCL have produced reliability coefficients higher than what we present here. For example in Mexican children, the test-retest reliability of the CBCL's scales ranged from 0.69 to 0.86 and internal consistency from 0.69 to 0.96 [29]. In China the test-retest reliability of the Internalising, Externalising, Attention and Total Problems was between 0.79 to 0.84 [30]. Larger studies are needed to confirm the reliability of the CBCL in Ugandan children as the current reliabilities are lower than those reported from similar studies elsewhere, possibly owing to the long test-retest interval (9 weeks) and small sample (N = 22).

Exceedingly low test-retest and internal reliabilities were observed for Thought Problems which contains items dealing the with child's covert behaviour (eg Hears things, Has strange ideas). Parents may find it difficult to be consistent in rating these covert behaviours which are not easily observed compared to the overt behaviours. In this study, low reliabilities (below 0.4) were mostly found in the covert behaviours while high reliabilities (above 0.7) were mainly in the overt behaviours.

Depressive symptoms and Thought Problems (a combination of depressive, obsessive-compulsive, hallucinatory and sleep problems) were commonly reported in the current study, similar to an earlier study among Ghanaian adults that found higher scores of anxiety and depressive symptoms among those with a history of malaria compared to controls who had never had the illness [31]. However the Ghanaian study was carried out in adults who had uncomplicated malaria while the current study is in children who had complicated malaria. The similarity in findings may thus be a coincidence subject to further investigation. Cerebral malaria has been associated with increased behavioural problems in African children [12,15,16]. It is therefore likely that the frequency of behavioural problems reported here are higher than in the general population of Ugandan children of similar age with no history of cerebral malaria or other central nervous system infection or disorder.

Ugandan children had higher scores than Group 3 children for most of the behavioural problems except attention problems (both Attention Problems in the Syndrome Scale and ADH Problems in the DSM Scale). This observation of more behavioural problems is not a surprise as prior studies have associated cerebral malaria with behavioural problems in children [13-16]. Consistent with the low frequency of attention problems, Cohen's *d *for both attention problems was below zero indicating that the Group 3 children had higher scores on attention problems than the Ugandan children. However caution is needed when interpreting our findings due to lack of Ugandan norms for the present study and the limited sample size. Lack of Ugandan norms makes it difficult to confidently conclude whether the observed problems are due to cerebral malaria, environmental characteristics, problems with the translation or a combination of factors.

A high frequency of attention deficits was earlier observed in these children [11] which is contrary to the present findings. This could be attributed to the different methods of assessing attention in the studies. John and colleagues [11] used a computerised measure of attention based on the child's ability to respond to the target stimuli [32] while the present study used parents' endorsement of behaviour depicting attention problems. The computerised method is a measure of sustained attention measuring the child's reaction time in milliseconds and the ability to discriminate between a target and non target [32]. Sustained attention is best measured by computerised tests [33] as used in the earlier study documenting attention problems [11]. The CBCL on the other hand gives a broad description of the child's behaviour and may not accurately measure sustained attention like the computerised tests. When the CBCL attention scores of children in the current study who were earlier categorised as having attention impairment [11] were compared to those not impaired, there was no significant difference in the scores (data not shown) which may suggest that these two tests may not measure the same kind of attention. We cannot fully explain this finding of lower attention problems measured by the CBCL, further studies are needed before conclusive statements can be made.

This under reporting of attention problems by the Ugandan parents may partly explain why the Internalising and Externalising Scales had a higher frequency of children with problems of clinical importance compared to the Total Problems Scale. The Ugandan parents consistently rated highly the behaviours making up the Internalising and Externalising Scales than the Group 3 parents but this was reversed for the attention problems.

This study was not able to evaluate the CBCL's concurrent validity since no other measures of child behaviour were administered due to time constraints. In addition, our sample was not recruited from a psychiatric setting where their clinical diagnoses could be compared with the CBCL scores to evaluate its sensitivity and specificity. A further limitation of our study was the limited sample size of 64 children which gave a small number of 22 for the test-retest reliability calculation. With the nine week interval between the baseline and post-intervention testing, it can be argued that important changes in behaviour can take place. This we believe was not the case in this study as comparison of the two test scores of the control group showed no significant changes [26].

## Conclusion

The Luganda version of the CBCL has moderate reliability and can be used in behavioural assessment. Depressive and thought problems are likely behavioural outcomes of cerebral malaria. Future studies are needed to document these problems and their course, develop country norms for the CBCL, evaluate its validity in a clinical sample so as to determine its sensitivity and specificity and provide a broader range of responses.

## Competing interests

The authors declare that they have no competing interests.

## Authors' contributions

PB participated in the design of the study, enrolment of participants, carried out the statistical analyses, wrote the manuscript and approved the final version for publication. NN participated in the design of the study, wrote the manuscript and approved the final version for publication. BG conceived the study, participated in the design of the study, wrote the manuscript and approved the final version for publication, ROO participated in the design of the study, enrolment of participants, wrote the manuscript and approved the final version for publication. CCJ participated in the design of the study, wrote the manuscript and approved the final version for publication. MJB conceived the study, participated in the design of the study, wrote the manuscript and approved the final version for publication.
